# Editorial: Stem Cells as Targeted Drug Delivery Vehicles

**DOI:** 10.3389/fphar.2020.614730

**Published:** 2020-12-21

**Authors:** Gina D. Kusuma, Jessica E. Frith, Christopher G. Sobey, Rebecca Lim

**Affiliations:** ^1^ The Ritchie Centre, Hudson Institute of Medical Research, Melbourne, Australia; ^2^ Department of Obstetrics and Gynaecology, Monash University, Melbourne, Australia; ^3^ Department of Materials Science and Engineering, Monash University, Melbourne, Australia; ^4^ Department of Physiology, Anatomy & Microbiology, School of Life Sciences, La Trobe University, Melbourne, Australia

**Keywords:** stem cells, paracrine, drug delivery, bioengineering, extracellular vesicles

**Editorial on the Research Topic**

**Stem Cells as Targeted Drug Delivery Vehicles**

The therapeutic benefits of many stem cell-based therapies are now widely believed to be mediated through the secretion of paracrine factors, such as cytokines, chemokines, growth factors, and extracellular vesicles (EVs). The future of stem cell-based therapies may well lie in our ability to manipulate these factors. As stem cells are sensitive to their microenvironment, the components of their secretome may be manipulated by altering their culture conditions. This themed issue comprises narrative reviews and original research articles on the emerging therapeutic use of stem cells and EVs, along with novel bioengineering and manufacturing technologies that leverage these paracrine outputs.

## Stem Cells as Drug Factories for Therapeutic Factors

The therapeutic benefit of mesenchymal stem/stromal cell (MSC)-based therapies has been attributed to their pleiotropic effects, mainly through their secretome including EVs, which are nanovesicles secreted by all cells to facilitate intercellular communication. A significant hurdle to widespread use of EVs lies in the ability to manufacture them at scale. Zavala et al. describe a method for efficient enrichment of MSC-derived EVs by encapsulating the MSCs in semi-permeable cellulose beads. By creating capsules of a pre-specified pore size, the authors were able to selectively control the release of particles less than <200 nm in diameter. This was achieved while maintaining the MSCs in 3D culture and producing EVs with characteristics comparable to those derived from standard 2D MSC cultures. This method also retained the *in vitro* biological properties of MSCs, including angiogenesis, immunosuppression and stimulation of neuritic outgrowth (Zavala et al.).

## Bioengineering Cellular Microenvironment to Modify Paracrine Function

The microenvironment is a critical contributor to the paracrine function of MSCs. Brooks et al. compared *ex vivo* immunomagnetic bead-sorted adipose tissue-derived mesenchymal stromal cells (ASCs) against culture-expanded ASCs. They reported significant changes to global gene expression during the first 3 days of culture where *ex vivo*-sorted ASCs more readily differentiated into mesenchymal lineages. The levels of paracrine molecules were also significantly different (Brooks et al.), suggesting that even a brief culture period could affect ASC characteristics.

## Next Generation Scalable Manufacturing of Stem Cells

A major bottleneck in the industrialisation of stem cell manufacturing includes the scalable expansion of stem cells while maintaining stem cell phenotype and fidelity of potency. Cherian et al. summarised the current commercial manufacturing solutions for MSCs and their impact on bioactivity and their secretome. The contribution of substrate stiffness, surface topography and extracellular matrix components to microenvironmental cues for MSCs expansion were discussed (Cherian et al.).

## Nanoparticles in Regenerative Medicine

While MSC-derived EVs show great potential for therapeutic applications, their uptake mechanisms remain poorly understood. Huang et al. describes an MSC-EV uptake mechanism involving common endocytosis in monocytes and keratinocytes, mediated by heparan sulfate proteoglycans on cell surfaces. Osteogenic, chondrogenic, and adipogenic EVs induced significant increases in the expression levels of respective lineage-specific marker genes in recipient cells and these effects were verified *in vivo* using a mouse subcutaneous implantation model (Huang et al.). These findings show the opportunity to modulate EV cargo and direct tissue-specific regeneration using EVs from differentiated MSCs.

Riau et al. discusses the challenges of conventional EV administration and highlights key techniques in fabricating novel sustained delivery systems for EVs in their mini review. Biodegradable hydrogels used to encapsulate EVs prevent premature clearance and allow a more concentrated EV dose at the target site. This EV-hydrogel system has been used to stimulate skin regeneration, angiogenesis, cardiac regeneration and wound healing (Riau et al.).

On a similar note, Shukla et al. summarises the clinical applications of adipose tissue including contemporary attempts to enrich ADSCs within the fat graft, harnessing paracrine effects of the ADSC secretome, and the most recent iteration—ADSC-derived EVs. Components of the ADSC secretome have been shown to promote wound healing and neuro-regeneration, ameliorate renal diseases, and for cardiac protection (Shukla et al.).

Golinelli et al. discusses the potential of MSC-based anti-cancer strategies. Due to the ability of MSCs to engraft into malignant tissues and their immune-privileged status, the authors postulate that MSCs may be ideal vehicles for the delivery of anti-cancer agents. Their review summarises the two major strategies in using MSCs to target cancer. Firstly, the non-genetic modification of MSCs by loading chemotherapeutic agents (via nanoparticles and/or EVs) for targeted delivery at tumor sites, and secondly, the genetic modification of MSCs to induce the expression of anticancer proteins, oncolytic viruses or suicide genes. The potential for MSC-based therapies in oncology lies in the combination of tumour-targeting approaches to improve MSC homing (Golinelli et al.).

In a tumor microenvironment, cancer stem cell (CSC)-EVs mediate cell-to-cell communication to support and promote tumorigenesis, where alterations to parent cells will also alter EV secretion. The opinion paper by Al-Sowayan et al. discusses the increased awareness of CSCs and highlights their possible role in promoting cancer progression by facilitating metastasis. Studies that target CSCs and inhibition of CSC-EV release and/or uptake may be an impetus for anti-cancer drug development (Al-Sowayan et al.).

## Novel Biomaterials for Stem Cells Delivery

Mukherjee et al. investigated the use of tissue-engineered constructs for pelvic organ prolapse repair, on the basis that biomimetic and biodegradable nanofiber meshes mimicking natural ECM would yield superior vaginal constructs by reducing the foreign body response. The implanted constructs resulted in significantly increased expression of genes associated with ECM regulation, cell adhesion, angiogenesis, and immune response compared to the nanomesh alone. The combination of biomaterials and endometrial MSCs reduced acute inflammation and showed the hallmarks of successful implantation (Mukherjee et al.).

The breadth of articles covered within this Research Topic demonstrate the diversity in stem cell and EV research, as well as the significant challenges for clinical translation. The development of stem cells as drug delivery vehicles is rapidly progressing and the translational effort will require collaboration between multidisciplinary experts (stem cell biologists, biomaterial scientists, bioengineers, regulatory experts, healthcare professionals) involved in all development stages of stem cell-derived products including discovery research, manufacturing, preclinical and clinical trials.

## Author Contributions

GK and RL wrote this article. JF and CS have made a direct and intellectual contribution to the work. All authors have approved the article for publication.

## Conflict of Interest

The authors declare that the research was conducted in the absence of any commercial or financial relationships that could be construed as a potential conflict of interest.

